# L^59^ TGF-β LAP degradation products serve as a promising blood biomarker for liver fibrogenesis in mice

**DOI:** 10.1186/s13069-015-0034-9

**Published:** 2015-09-15

**Authors:** Mitsuko Hara, Ikuyo Inoue, Yuta Yamazaki, Akiko Kirita, Tomokazu Matsuura, Scott L. Friedman, Daniel B. Rifkin, Soichi Kojima

**Affiliations:** Micro-Signaling Regulation Technology Unit, RIKEN Center for Life Science Technologies, Wako, Saitama 351-0918 Japan; Department of Laboratory Medicine, The Jikei University School of Medicine, Minato-ku, Tokyo 105-8461 Japan; Division of Liver Diseases, Icahn School of Medicine at Mount Sinai, New York, NY 10029 USA; Department of Cell Biology, New York University School of Medicine, New York, NY 10016 USA; Department of Medicine, New York University School of Medicine, New York, NY 10016 USA

**Keywords:** Biomarker, Hepatic stellate cells, Fibrogenesis, Liver fibrosis, Plasma kallikrein (PLK), TGF-β activation

## Abstract

**Background:**

Hepatic fibrosis, which is the excessive accumulation of extracellular matrices (ECMs) produced mainly from activated hepatic stellate cells (HSCs), develops to cirrhosis over several decades. There are no validated biomarkers that can non-invasively monitor excessive production of ECM (i.e., fibrogenesis). Transforming growth factor (TGF)-β, a key driver of fibrogenesis, is produced as an inactive latent complex, in which active TGF-β is enveloped by its pro-peptide, the latency-associated protein (LAP). Thus, active TGF-β must be released from the complex for binding to its receptor and inducing ECM synthesis. We recently reported that during the pathogenesis of liver fibrosis, plasma kallikrein (PLK) activates TGF-β by cleavage between R^58^ and L^59^ residues within LAP and that one of its by-products, the N-terminal side LAP degradation products ending at residue R^58^ (R^58^ LAP-DPs), can be detected mainly around activated HSCs by specific antibodies against R^58^ cleavage edges and functions as a footprint of PLK-dependent TGF-β activation. Here, we describe a sandwich enzyme-linked immunosorbent assay (ELISA) that detects the other by-products, the C-terminal side LAP-DPs starting from residue L^59^ (L^59^ LAP-DPs). We demonstrated that the L^59^ LAP-DPs are a potentially novel blood biomarker reflecting hepatic fibrogenesis.

**Results:**

We established a specific sandwich ELISA to quantify L^59^ LAP-DPs as low as 2 pM and measured L^59^ LAP-DP levels in the culture media of a human activated HSC line, TWNT-4 cells. L^59^ LAP-DPs could be detected in their media, and after treatment of TWNT-4 cells with a TGF-β receptor kinase inhibitor, SB431542, a simultaneous reduction was observed in both L^59^ LAP-DP levels in the culture media and the mRNA expression levels of *collagen type* (*I*) *α1*. In carbon tetrachloride- and bile duct ligation-induced liver fibrosis models in mice, plasma L^59^ LAP-DP levels increased prior to increase of hepatic hydroxyproline (HDP) contents and well correlated with α-smooth muscle actin (αSMA) expression in liver tissues. At this time, αSMA-positive cells as well as R^58^ LAP-DPs were seen in their liver tissues.

**Conclusions:**

L^59^ LAP-DPs reflect PLK-dependent TGF-β activation and the increase in αSMA-positive activated HSCs in liver injury, thereby serving as a novel blood biomarker for liver fibrogenesis.

**Electronic supplementary material:**

The online version of this article (doi:10.1186/s13069-015-0034-9) contains supplementary material, which is available to authorized users.

## Background

Hepatic fibrosis, the common pathology resulting from chronic liver diseases regardless of etiology, is characterized as the excessive deposition of extracellular matrices (ECMs) produced mainly from activated hepatic stellate cells (HSCs) in injured tissue [1, 2]. Because early fibrosis is often asymptomatic, yet steadily progresses toward cirrhosis, it is important to monitor fibrogenic activity both to track disease progression and eventually to assess response to anti-fibrotic drugs. Liver biopsy, a widely accepted technique, is highly invasive and reflects only already-accumulated ECMs, but not ongoing ECM synthesis. Thus, a non-invasive marker for fibrogenesis in the liver is a major unmet need in the field [3, 4].

A key driver of liver fibrosis is transforming growth factor (TGF)-β [5]. TGF-β is produced as a homodimeric precursor protein consisting of an N-terminal pro-region named latency-associated protein (LAP) and a C-terminal region that becomes the biologically active TGF-β molecule. After processing at R^278^-A^279^ by a furin-like protease, the cleaved LAP still remains non-covalently associated with the active TGF-β, forming a small latent complex and preventing active TGF-β from binding to its cognate receptors. Therefore, active TGF-β must be released from the latent complex, a process called TGF-β activation, to induce ECM production [6, 7]. Resultant active TGF-β binds to cognate serine/threonine kinase signaling receptors and via the Smad signaling pathway stimulates the expression of target genes, including ECM proteins and TGF-β itself, in an autocrine manner [5, 8].

We recently found that plasma kallikrein (PLK) cleaves LAP between R^58^ and L^59^ residues to cause TGF-β1 activation. We further demonstrated that this mechanism underlies hepatic fibrosis in animal models, as well as in patients, by detecting the N-terminal side LAP degradation products (…QILSKLR^58^) ending at R^58^ residue (R^58^ LAP-DPs) in fibrotic livers using a specific antibody we generated [9]. R^58^ LAP-DPs are S-S bonded to latent TGF-β binding protein via C^33^ residue. These DPs were detected primarily around α-smooth muscle actin (αSMA)-positive activated HSCs, both in peri-sinusoidal fibrotic regions prior to excessive ECM deposition, suggesting that R^58^ LAP-DPs might be a marker that reflects PLK-dependent TGF-β activation [9]. On the other hand, the C-terminal side LAP-DPs (L^59^ASPPSQ…), beginning from L^59^ residue (L^59^ LAP-DPs), was not detectable in the liver tissues, perhaps because L^59^ LAP-DPs might be released into the blood.

Here, we describe a sandwich enzyme-linked immunosorbent assay (ELISA) using the specific antibody against L^59^ LAP-DPs and examine whether L^59^ LAP-DPs can be used as a blood biomarker reflecting PLK-dependent TGF-β activation that correlates with activated HSCs and whether they can be used to monitor liver fibrogenesis in mice.

## Results

### Establishment of a sandwich ELISA for measuring L^59^ LAP-DPs

**Fig. 1:**
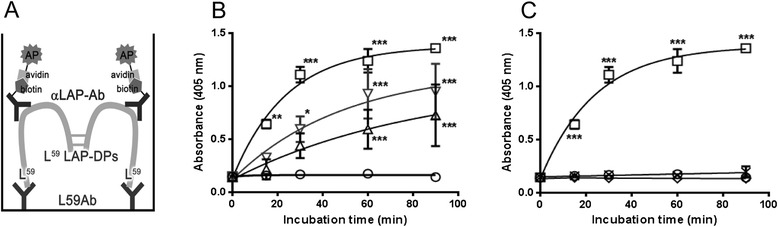
Establishment of a sandwich ELISA for detecting L^59^ LAP-DPs. **a** Schematic diagram of the ELISA for L^59^ LAP-DPs. In the ELISA, L^59^ LAP-DPs are first captured by coated L59 antibodies (Ab) and then sandwiched by biotin-conjugated anti-LAP antibodies (αLAP-Ab), which form a complex with strep-AP. For detection, an enzyme substrate is added and absorbance at 405 nm was measured. **b**, **c** Incubation time- and PLK concentration-dependent increases in the absorbance of the samples containing LAP incubated with PLK. The LAP (final 25 nM) was digested with 12.5 nM (open triangle), 25 nM (open reverse triangle), and 50 nM (open square) PLK, or without PLK (open circle) for 0–90 min, and then the samples were diluted by 1/50 and subjected to the L^59^ LAP-DP ELISA (**b**). Also, 25 nM rhLAP was digested with 50 nM PLK (open square) or PLN (cross mark), or PLK in the presence of 5 μM camostat mesilate (open diamond) (**c**). All data were presented as mean ± SD from two different experiments ​*p-value <0.05, **p-value <0.01, ***p-value<0.001 obtained comparing to corresponding control values

To quantify the levels of L^59^ LAP-DPs in a culture medium of collagen-producing cells including HSCs or blood from animal models with fibrogenesis, we established a sandwich ELISA using a combination of L59 and commercially available anti-LAP antibodies (Fig. 1a) and examined the specificity of the ELISA for L^59^ LAP-DPs initially in test tube reactions. The absorbance of a sample containing recombinant human LAP β1 (rhLAP β1) incubated with PLK, in which L^59^ LAP-DPs are produced, increased proportionately to both the duration of PLK digestion and the concentration of PLK (Fig. 1b). This increase was completely abolished in the presence of a protease inhibitor, camostat mesilate (Fig. 1c, open diamond). In addition, there was no increase in the absorbance in the sample containing LAP incubated with plasmin (PLN) (Fig. 1c, cross mark), in which LAP was degraded between K^56^ and L^57^ residues [9, 10]. These results suggest that the established ELISA is specific for L^59^ LAP-DPs and may reflect PLK-dependent TGF-β activation reaction in vitro, as we reported for the R58 antibody [9]. We made the standard curve by diluting standard L^59^ LAP-DPs obtained after incubating rhLAP β1 with an equal amount of PLK at 37 °C for 2 h. By a Western experiment, we confirmed that rhLAP β1 was almost completely digested to L^59^ LAP-DPs under this condition (data not shown). The absorbance linearly increased up to 100 pM (Additional file 1: Figure S1). We were able to measure a minimum concentration of L^59^ LAP-DPs as low as 2 pM. From results of the spike and recovery test, we decided to dilute samples at least by 1:2 in the case of culture media and at least by 1:5 in the case of mouse plasma (Table. 1). Actually, we diluted plasma samples 1:10 in the following experiments. The recovery rate of diluted samples was 91 % (in average) both in culture media and in mouse plasma (Table 2). The mean variations among intra- and inter-assays were 3.9 ± 0.4 % (*N* = 12) and 9.8 ± 5.3 % (*N* = 10) in culture media and 8.7 ± 3.6 % (*N* = 6) and 6.5 ± 5.3 % (*N* = 6) in mouse plasma, respectively. Finally, analytes were stable at least after two freeze–thaw cycles for both culture media and mouse plasma.

**Table 1:** Summarized results of spike and recovery tests

Sample	Added spike (pM)	Dilution rate	Expected (pM)	Observed (pM)	% recovery	
					Average	Range
Culture media	50 pM	Neat	46	32	69.8	69–71
		1:2	46	40	*85.8*	85–87
		1:4	46	42	*91.9*	85–87
		1:8	46	44	*95.1*	93–98
Mouse plasma	200 pM	Neat	189	132	69.8	65–75
		1:5	201	180	*89.6*	65–75
		1:10	193	184	*95.5*	92–99
		1:20	190	197	*103.5*	103–104
	50 pM	Neat	47	29	61.8	58–66
		1:5	46	38	*82.7*	82–84
		1:10	46	40	*88.7*	88–89
		1:20	49	43	*88.7*	87–89

*Values exceeding 80 % are presented*

**Table 2:** Summarized results of linearity tests

Sample	Added spike (pM)	Dilution rate	Expected (pM)	Observed (pM)	% recovery	
					Average	Range
Culture media	50 pM	1:2	46	40	85.8	85–87
		1:4	46	42	91.9	90–94
		1:8	46	44	95.1	93–98
		Average 90.9 ± 4.7				
Mouse plasma	200 pM	1:5	201	180	89.6	65–75
		1:10	193	184	95.5	92–99
		1:20	190	197	103.5	103–104
	50 pM	1:5	46	38	82.7	82–84
		1:10	46	40	88.7	88–89
		1:20	49	43	88.7	87–89
		Average 91.3 ± 7.2				

**Fig. 2:**
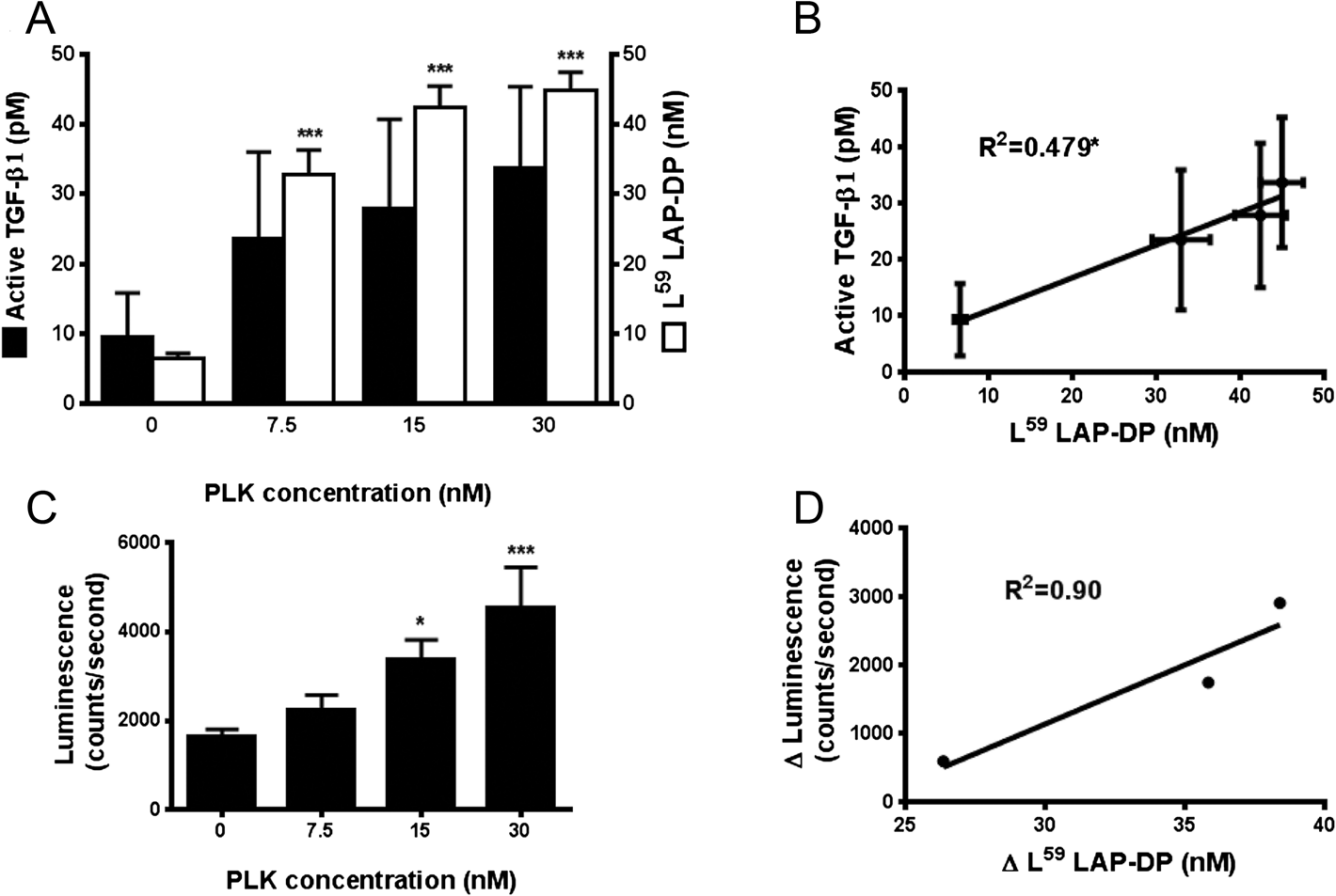
Correlation among L^59^ LAP-DPs and active TGF-β in the culture medium, and intracellular signal transduction. **a**, **c** The ×9CAGA-Luc-transformed CCL64 cells were cultured in PLK-added CM derived from HEK293T cells overexpressing hLTGF-β1. After 6 h, the levels of active TGF-β1 and L^59^ LAP-DPs were determined by respective ELISAs (**a**), and the extent of TGF-β signaling was measured by luciferase activity in CCL64 cells (**c**). **b**, **d** The scatterplots between the levels of L^59^ LAP-DPs and active TGF-β shown in **a** (**b**) and between increases in L^59^ LAP-DP levels and increases in luminescence from the values obtained compared to basal levels in the absence of PLK shown in **c** (**d**). A significant positive correlation was seen (**b**) *p-value <0.05, ***p-value <0.001 obtained comparing to 0

Using the ELISA, we examined whether L^59^ LAP-DPs might reflect TGF-β activation and therefore the bioactivity of the resultant active TGF-β. Mink lung epithelial cells stably transfected with a TGF-β-responsive reporter gene (×9 CAGA-Luc-transformed CCL64 cells) were incubated with human latent TGF-β1 (hLTGF-β1) in the presence or absence of PLK. This cell line produced negligible levels of endogenous LTGF-β1, active TGF-β1, and L^59^ LAP-DPs. As a source of hLTGF-β1, we used the conditioned media (CM) of HEK293T cells transiently transfected with an hLTGF-β1 construct. We incubated the ×9 CAGA-Luc-transformed CCL64 cells with the HEK293T cell CM containing 20 nM LTGF-β1, 10 pM active TGF-β1, and 5 nM L^59^ LAP-DPs. To assess TGF-β1 activation, namely, generation of active TGF-β1, we determined levels of both active TGF-β1 and L^59^ LAP-DPs in the culture media by respective ELISAs. At the same time, to assess the relationship between L^59^ LAP-DPs and cellular signals evoked by the resultant active TGF-β, we also measured increases in the luciferase activity in lysates made from the ×9 CAGA-Luc-transformed CCL64 cells. The levels of active TGF-β1 and L^59^ LAP-DPs increased depending on PLK concentration maintaining a ratio of 1:1300 (Fig. 2a). L^59^ LAP-DP levels also correlated with active TGF-β1 levels (Fig. 2b). Moreover, the luciferase activity in the lysates of the ×9 CAGA-Luc-transformed CCL64 cells also increased and correlated with L^59^ LAP-DP levels (Fig. 2c, d). These data suggested that L^59^ LAP-DPs reflected the activation of TGF-β and the bioactivity of the resultant active TGF-β in cells.

### L^59^ LAP-DP levels reflect fibrogenic activity of HSCs in vitro

**Fig. 3:**
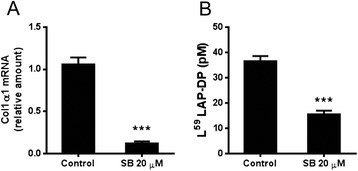
L^59^ LAP-DP levels in the culture media of TWNT-4 treated with SB431542. The (**a**) as well as  (**b**) were measured after a 72-h treatment of TWNT-4 with 20 μM SB431542 (SB). Data are mean ± SD (*N* = 3). Representative results from two different experiments with similar results are shown ***p-value <0.001

We examined whether L^59^ LAP-DP levels might reflect collagen production using an activated human HSC line, TWNT-4, that constitutively produces collagen [11]. The levels of active TGF-β1 in the media were quite low, less than the detection limit of the assay (~pM), while endogenously detected total TGF-β1 and L^59^ LAP-DPs were present at concentrations as high as 100 pM and 20–40 pM, respectively. After treatment of TWNT-4 cells with 20 μM SB431542 (a TGF-β signaling inhibitor [12]) for 72 h, ​we measured L^59^ LAP-DP levels in the culture media (Fig. 3b). When *Col Iα1* mRNA levels decreased to 1/30, L^59^ LAP-DP levels were lowered by 50 % and we failed to measure active TGF-β1 in the same culture media. These results suggested that active TGF-β1 disappeared from the culture media during the 72-h incubation after its generation, whereas its by-product, L^59^ LAP-DPs, remained detectable for at least 72 h after incubation, and their levels reflect fibrogenic activity of the activated HSCs.

### Correlation between plasma L^59^ LAP-DP levels and hepatic contents of hydroxyproline in animal models for liver fibrosis

**Fig. 4:**
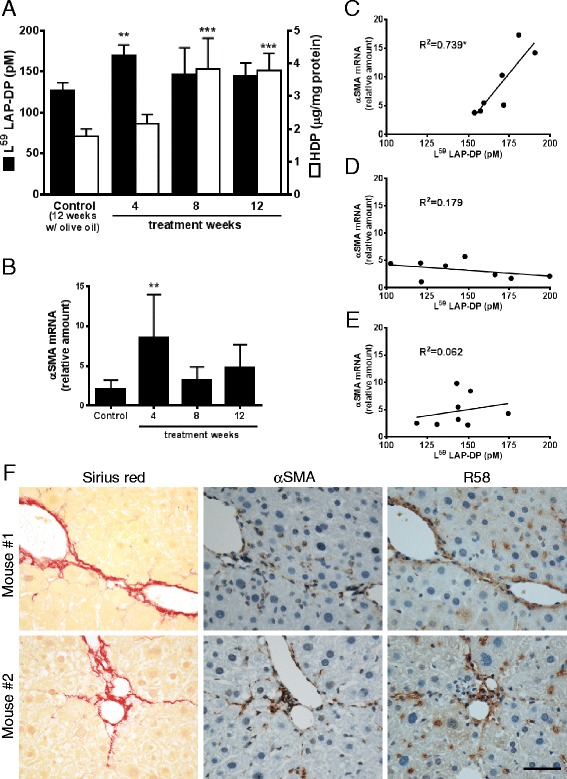
Plasma concentrations of L^59^ LAP-DPs in CCl_4_-treated mice. **a** Plasma concentrations of L^59^ LAP-DPs, as well as hepatic HDP contents, in CCl_4_-treated mice (*N* = 7–8) were measured. **b** The expression levels of *αSMA* mRNA in the liver tissues from CCl_4_-treated mice (*N* = 7–8) were determined. **c**–**e** The scatter plots between the plasma concentration of L^59^ LAP-DPs (**a**) and expression level of *αSMA* mRNA (**b**) in each mouse at 4 weeks (**c**), 8 weeks (**d**), and 12 weeks (**e**) after starting CCl_4_ treatment. **f** Immunostaining of liver sections from mice (*N* = 7–8) treated with CCl_4_ for 4 weeks. Fibrotic regions were stained by picro-sirius red (*left panels*). Their proximal sections were immunostained with anti-αSMA *(middle panels*) and R58 antibodies (*right panels*). Representative areas from two randomly selected mice were shown ​*p-value <0.05, **p-value <0.01, ***p-value <0.001 obtained comparing to control value

**Fig. 5:**
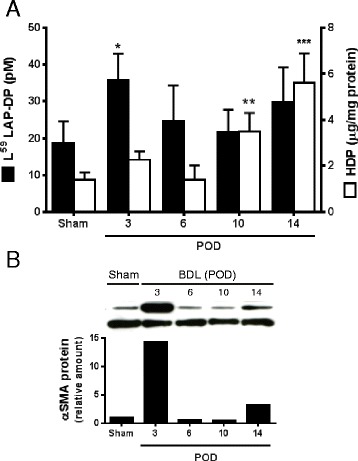
Plasma concentrations of L^59^ LAP-DPs in BDL mice. **a** Plasma concentrations of L^59^ LAP-DPs, as well as hepatic HDP contents in BDL mice, were measured at the indicated post-operative days (POD). **b** The expression of αSMA protein in liver tissues was examined by Western blotting. Expression levels were semi-quantitated using NIH imageJ. A set of experiments was performed using two to four mice in each group. Representative results from two different experiments with similar results are shown *p-value <0.05, **p-value <0.01, ***p-value <0.001 obtained comparing to sham

Next, we examined if plasma levels of L^59^ LAP-DPs might reflect hepatic fibrogenesis in vivo using two fibrotic animal models (Figs. 4 and 5). We determined the plasma concentrations of L^59^ LAP-DPs in carbon tetrachloride (CCl_4_)-treated mice (Fig. 4a, closed columns) and compared them with corresponding hepatic hydroxyproline (HDP) content, which reflects the amount of accumulated collagen or the extent of liver fibrosis (Fig. 4a, open columns). After starting CCl_4_ treatment, plasma L^59^ LAP-DP concentrations were significantly higher than in control mice at 4 weeks, before hepatic HDP content increased (Fig. 4a). At 4 weeks, *αSMA* mRNA expression increased (Fig. 4b), and the levels of plasma L^59^ LAP-DP correlated well with the levels of *αSMA* mRNA expression in each individual (Fig. 4c). On the other hand, there was no correlation between plasma L^59^ LAP-DP and *αSMA* mRNA expression at 8 and 12 weeks (Fig. 4d, e, respectively). As shown in Fig. 4f, excessive collagen fibers started to accumulate at 4 weeks after initiating CCl_4_ treatment. The αSMA-positive cells appeared, and signals of R^58^ LAP-DPs were also detectable at this time. A similar result was obtained in the mouse bile duct ligation (BDL) model (Fig. 5). Plasma L^59^ LAP-DP concentrations in BDL mice were higher than those in the control animals from post-operative day (POD) 3 to POD 14, whereas hepatic HDP levels gradually increased for up to POD 14. There was no obvious correlation between plasma L^59^ LAP-DP levels and the amounts of hepatic HDP at each mouse (Fig. 5a). On the other hand, pre-fibrotic animals at POD3, in which plasma L^59^ LAP-DP levels were significantly higher, showed a robust increase of αSMA protein expression in liver tissues (Fig. 5b). These results indicated that plasma L^59^ LAP-DP levels reflect ongoing fibrogenesis by the activated HSCs prior to excessive collagen accumulation, rather than measuring previously accumulated fibrosis.

## Discussion

In the present study, we have established a sandwich ELISA using the L59 antibody against the N-terminus of the L^59^ LAP-DPs (Fig. 1) and successfully detected L^59^ LAP-DPs in both human HSC cultures (Fig. 3) and in two mouse hepatic fibrosis models (Figs. 4 and 5). Our ELISA is specific for L^59^ LAP-DPs because it can specifically detect L^59^ LAP-DPs but not non-digested LAP and PLN-generated L^57^ LAP-DPs. The result of a protein-protein Blast (blastp) search showed that only myostatin/GDF8 has a partially similar cleavage sequence with the N-terminal side sequence (…Q^60^ILSKL**RL**ETAP…) [7]. However, we confirmed that the L59 as well as LAP antibodies do not recognize GDF8 incubated with PLK (data not shown).

When ×9 CAGA-Luc-transformed CCL64 cells were cultured with HEK293T cell CM and PLK, we observed parallel increases in L^59^ LAP-DPs, active TGF-β1 levels, and resultant luciferase activities (Fig. 2). However, the level of active TGF-β1 (~33 pM) was 1300 times lower than that of L^59^ LAP-DPs (~43 nM). Because the CM contained about 20 nM dimeric LTGF-β1, active TGF-β1 (~33 pM) was quite small (only 0.2 %). It is well known that TGF-β is very sticky and easily adheres to the surface of culture dishes, so levels in the culture media decrease rapidly. Furthermore, in vivo, active TGF-β is quickly cleared from the circulation (T1/2 = 1.6 ± 0.71 min) [13], thereby making it is difficult to measure accurate levels. We determined that T1/2 of L^59^ LAP-DPs is about 8 h (Additional file 2: Figure S2). This T1/2 value is comparable to that of aspartate aminotransferase, a well-known blood marker for liver injury [14]. The longer T1/2 value for L^59^ LAP-DPs compared to active TGF-β1 affords significant advantages in terms of being used as a non-invasive biomarker of fibrogenesis.

We speculated that L^59^ LAP-DPs were gradually lost due to either trapping by other proteins or further degradation by other proteases to eliminate their antigenic activity. We are now trying to specify the molecular species detected with our ELISA.

We failed to detect L^59^ LAP-DPs in primary rat HSC cultures, while we did detect them in both the culture media of human HSC line, TWNT-4, and in rat plasma. As we detected almost the same amounts of LTGF-β1 in the CM of activated rat primary HSCs and TWNT-4, we speculate that LAP-DP levels should be similar. In support of this possibility, we observed an almost similar extent of R^58^ LAP-DP staining in the cultures of activated rat primary HSCs and TWNT-4 (data not shown). Nevertheless, we failed to detect L^59^ LAP-DPs in the culture medium of activated rat primary HSCs. We speculate that the reasons could include the superior reaction specificity to human L^59^ LAP-DPs than rat L^59^ LAP-DPs as well as the lower concentration of L^59^ LAP-DPs in the culture media compared to those in rat blood (plasma). In vitro, we cultured 3 × 10^5^ rat HSCs using 2 ml culture medium, whereas in vivo, a rat has 5–12 × 10^7^ HSCs in the liver and 20 ml blood [15], suggesting that since a rat HSC produces the same amounts of L^59^ LAP-DPs both in vitro and in vivo, their concentrations in the culture media should be 1/20–40 of those in blood (namely, 1/40–80 in plasma), which is below the detection limit of the assay. We attempted to concentrate the culture medium; however, the concentrated contaminant proteins, mostly bovine serum albumin (BSA), interfered with the performance of the ELISA. As a result of these limitations, we could not use primary rat or mouse HSCs. Instead, we used TWNT-4 cells and demonstrated that the levels of L^59^ LAP-DPs in the media correlated with collagen production. A simultaneous reduction was seen between levels of collagen mRNA and L^59^ LAP-DPs in SB431542-treated cells, suggesting that L^59^ LAP-DP levels reflect fibrogenesis in vitro.

In the CCl_4_ and BDL models, the plasma levels of L^59^ LAP-DPs significantly increased at the early stage of fibrosis prior to excessive collagen accumulation, when αSMA expression is robustly increased in the liver. At this time, there was a good correlation between the L^59^ LAP-DP levels in the plasma and αSMA expression in the liver. It was demonstrated that the expression of pro-fibrogenic genes oscillated and decreased after robust increase at the early stage, and thereafter increased again at the later stage [16–18]. Supporting these reports, plasma L^59^ LAP-DPs levels as well as αSMA expressions decreased and were not significantly higher in fibrotic mice than in control mice after the early stage of fibrosis (Figs. 4 and 5). These data suggest that the L^59^ LAP-DPs reflect fibrogenesis by the αSMA-positive cells and might be a predictive marker for fibrogenic activity and thus the accumulation of fibrosis at the early stage of fibrosis. On the other hand, the L^59^ LAP-DP levels in the plasma did not correlate with αSMA expression in the liver at the late stage, and imply that PLK-dependent TGF-β activation mainly contributes to earlier fibrosis. Integrin β6 transcripts are reportedly up-regulated and correlate with the progression/stage of fibrosis in both BDL and Mdr2^−/−^ biliary fibrotic mice [19], indicating that integrin-dependent TGF-β activation occurs in later fibrosis. These observations imply that TGF-β is activated by distinct molecular mechanisms at different stages of fibrosis.

Not only activated HSCs, but also portal fibroblasts, contribute to liver fibrosis [20, 21]. It will therefore be worthwhile to examine PLK-dependent TGF-β activation in portal fibroblasts in future studies.

Recently, Konuma et al. reported that R^58^ LAP-DPs were detected in melanocortin 4 receptor-deficient mice fed a high-fat diet, which exhibit steatohepatitis and fibrosis similar to human non-alcoholic steatohepatitis (NASH) [22]. In this model, positive reactivity for R^58^ LAP-DPs was found around hepatic crown-like structures (hCLS), in which macrophages surround dead or dying hepatocytes containing large lipid droplets. Moreover, both myofibroblasts and fibrosis were observed nearby in areas of hCLS and were reduced when fibrosis was improved by treatment with eicosapentaenoic acid [22]. This result indicated that PLK-dependent TGF-β activation also occurs in the pathogenesis of NASH-related fibrosis in rodents. We are now examining whether plasma L^59^ LAP-DP levels might increase in the high-fat diet-fed melanocortin 4 receptor-deficient mice and decrease in response to eicosapentaenoic acid. If this is the case, plasma L^59^ LAP-DP levels might be used for monitoring the therapeutic efficacy of anti-fibrotic agents in NASH patients.

Because our preliminary studies showed that plasma L^59^ LAP-DPs tend to be higher in patients suffering from hepatitis than in healthy volunteers, we are now investigating whether plasma L^59^ LAP-DPs can be used as a fibrogenesis blood marker in patients with chronic active liver disease.

## Conclusions

In summary, we have established a sandwich ELISA to detect by-products, L^59^ LAP-DPs, of PLK-dependent TGF-β activation using the L59 antibody against the N-termini of L^59^ LAP-DPs. In vitro, by treating hLTGF-β1 with PLK, L^59^ LAP-DPs were generated, and their levels correlate with both active TGF-β and *Col Iα1* mRNA levels. In in vivo CCl_4_ and BDL models, plasma L^59^ LAP-DP levels increase at the early stage of liver fibrosis prior to collagen deposition and correlate well with hepatic αSMA expression. These results suggest that plasma L^59^ LAP-DPs reflect PLK-dependent TGF-β activation and fibrogenesis in the liver and therefore may serve as a novel blood biomarker for hepatic fibrogenesis.

## Methods

### Materials

rhLAP β1 and anti-human LAP β1 mouse monoclonal antibodies were purchased from R&D Systems (Minneapolis, MN, USA). Streptavidin-conjugated alkaline phosphatase (strep-AP) was from Jackson ImmunoResearch Laboratories, Inc. (West Grove, PA, USA). Human PLK was from Calbiochem (San Diego, CA, USA), and human PLN, SB431542, and anti-αSMA antibody for Western blotting were from Sigma Chemical Co. (St. Louis, MO, USA). Anti-αSMA antibody for immunostaining was from Dako (Glostrup, Denmark). Camostat mesilate was purchased from Wako Pure Chemical Industries, Ltd. (Osaka, Japan).

### ELISAs

Active TGF-β1 was measured using a Promega TGF-β1 Emax Immuno Assay System ELISA kit (Promega Co., Madison, WI, USA). The sandwich ELISA for L^59^ LAP-DPs was established by combination of the specific antibody against L^59^ LAP-DP [9] and biotin-conjugated mouse monoclonal anti-LAP antibody (BAM2462, R&D Systems). The Nunc F96 Maxisoap Immunoplate (Thermo Fisher Scientific, Inc., Waltham, MA, USA) was coated with 20 μg/ml anti-L^59^ LAP-DP antibody in Tris-buffered saline (pH 7.3) at 4 °C overnight. After blocking with Tris-buffered saline containing 1 % BSA at 4 °C overnight, samples containing L^59^ LAP-DPs were added and incubated overnight at 4 °C, followed by sandwiching with 1 μg/ml biotin-conjugated anti-LAP antibody in HEPES buffer for 3 h at room temperature. After another 3 h of incubation with strep-AP, 4-nitrophenylphosphate disodium salt hexahydrate in diethanolamine buffer (pH 9.8) was added and absorbance at 405 nm was measured. The plates were washed by Tris-buffered saline containing 0.05 % Tween 20 between each step. For preparing L^59^ LAP-DP standards, rhLAP β1 (40 nM) was digested with PLK (40 nM) in 1 % BSA containing phosphate-buffered saline for 2 h at 37 °C.

### Evaluation of L^59^ LAP-DP ELISA

The lowest limit of quantification (LOQ) was defined as the value whose lower 2 SD value at absorbance at 405 nm did not overlap with the upper 2 SD value of the blank’s absorbance at 405 nm. We examined this for 12 times and choose the highest LOQ value. Certain amounts of the standard L^59^ LAP-DP stock were spiked into culture media and pooled mouse plasma, and then evaluated for the recovery and linearity of this ELISA. Distinct culture media (*N* = 10) and pooled mouse plasma (*N* = 6) containing different L^59^ LAP-DP concentrations were tested from three to six wells from one plate for intra-assay, and from three wells from two to three plates for inter-assay.

### Preparation of LTGF-β1 containing culture media

HEK293T cells (1 × 10^6^ cells) were seeded on a 60-mm dish. After 24 h, the pcDNA3 vector with an inserted human LTGF-β1 gene was transiently transfected to HEK293T cells using Lipofectamine 2000. After an additional 24 h, transfected HEK293T cells were cultured in DMEM containing 0.1 % BSA for another 24 h, and the CM were collected and used for the luciferase assay as described below.

### Luciferase assay

Mink lung epithelial cells stably transfected with a TGF-β-responsive reporter gene (×9 CAGA-Luc CCL64 cells) were seeded at 1 × 10^4^/well in a 96-well plate and cultured overnight [23]. After cells were treated with hLTGF-β1 in the absence or presence of PLK for 6 h, the luciferase activity in each cell lysate was measured by the Luciferase Assay System provided by Promega Co. (Madison, WI, USA).

### HSC culture

Human HSC (TWNT-4) cells [11] were cultured in the stellate cell medium purchased from ScienCell Research Laboratories (Carlsbad, CA, USA) at 37 °C in 5 % CO_2_. Cells were seeded at 4 × 10^4^/well in a 6-well plate, grown until 70 % confluence, and treated with 20 μM SB431542 for 72 h. The levels of L^59^ LAP-DPs in the culture media were measured by the ELISA developed in this study, and the mRNA levels in each cell lysate were quantitated by real-time reverse transcription-polymerase chain reaction (RT-PCR).

### Animals

Male C57BL/6 mice were purchased from Japan SLC, Inc. (Shizuoka, Japan). All animals were maintained on a 12-h light/12-h dark cycle. Food and water were available ad libitum. All animal experiments were performed in accordance with protocols approved by the RIKEN Institutional Animal Use and Care Administrative Advisory Committee.

### CCl_4_ model

Seven-week-old C57BL/6 mice (*N* = 7–8) were given repeated intramuscular injections of 50 % CCl_4_ (CCl_4_: olive oil = 1:1) (2 ml/kg twice a week). Control animals were injected with the same volume of olive oil. After 4, 8, and 12 weeks, the animals were sacrificed and samples of plasma (EDTA bleeding) and liver tissues were collected.

### BDL model

Ligation of the common bile duct was performed as described previously [24]. The common bile ducts of 8-week-old C57BL/6 mice (*N* = 2–4) were double-ligated and cut. The BDL animals were sacrificed on post-operative days 3, 6, 10, and 14. Sham-operated mice were treated in the same manner except that the bile duct was not ligated. Their samples were collected on post-operative day 14.

### Measurement of HDP content

HDP contents were measured as described previously [25].

### Staining of tissue sections

Animal tissue specimens were fixed in 4 % paraformaldehyde and embedded in paraffin. Liver sections (4-μm thickness) were stained by 0.1 % picro-sirius red [26] and immunostained by incubating overnight at 4 °C with anti-αSMA (diluted with 1:100) and R58 antibodies (1 μg/ml). EnVision/HRP (Dako, Glostrup, Denmark) was used as the second antibody and signal-amplifying system. The tissues were counterstained with hematoxylin.

### Quantitative real-time RT-PCT

The total RNA was extracted from HSCs and liver tissues using RNeasy Micro/Mini kits (Qiagen, Hilden, Germany). After synthesis of first-strand cDNA with the Prime Script RT kit (TaKaRa Bio., Japan), qPCR was performed with SYBR Premix (TaKaRa Bio., Japan) in the thermal cycler. Specific primers used are listed in Table 3. The results were normalized for *GAPDH* mRNA levels.

**Table 3:** Primers used in quantitative RT-PCR

Target	Primer sequences	
	5′-primer	3′-primer
human *GAPDH*	GGAGTCAACGGATTTGGT	AAGATGGTGATGGGATTTCCA
human *Col Iα1*	ACGAAGACATCCCACCAATC	AGATCACGTCATCGCACAAC
mouse *GAPDH*	AACTTTGGCATTGTGGAAGG	ACACATTGGGGGTAGGAACA
mouse *αSMA*	ACAGCCCTCGCACCCA	GCCACCGATCCAGACAGAGT

### Western blotting

Small pieces of liver tissue were homogenized with RIPA buffer (50 mM Tris–HCl [pH 7.5], 150 mM NaCl, 1 % Triton X, 1 mM EDTA, 10 % sucrose, and cOmplete, a protease inhibitor cocktail [Roche, Mannheim, Germany]). Homogenates were clarified by centrifugation at 18,000 rpm for 20 min at 4 °C, and the total protein concentration of each supernatant was determined using a BCA protein assay reagent kit (Thermo Fisher Scientific Inc., Rockford, IL, USA). Proteins (20 μg/lane) were separated by SDS-PAGE and transferred to a PVDF membrane (Millipore, Bedford, MA, USA). Western blot analyses were performed using either anti-αSMA and anti-GAPDH antibodies plus HRP-conjugated anti-mouse antibodies (1:5000) (Jackson Immuno Research Laboratories, Inc., West Grove, PA, USA). The bands were visualized by a Western Blotting Substrate Plus reagent purchased from Thermo Fisher Scientific (Rockford, IL, USA) and semi-quantitated using NIH imageJ.

### Statistical analysis

Quantitative data are shown as mean ± SD. Statistical analyses were performed using GraphPad Prism version 6.0 for Windows (GraphPad Software, San Diego, CA, USA). *p value <0.05, **p value <0.01, and ***p value <0.001 were considered as statistically significant.

## Additional files

Additional file 1: Figure S1. The standard curve for the L^59^ LAP-DP ELISA. The absorbance linearly increased up to about 100 pM. The lower limit of quantitation is approximately 2 pM. Additional file 2: Figure S2. The half-life of L^59^ LAP-DPs in mouse plasma. Exogenously generated L^59^ LAP-DPs were added to mouse plasma and incubated at 37 °C for 0, 1, 2, 4, 8, 16, and 24 h. The remaining levels were measured and plotted against incubation time, and the half-life time was determined.

## Abbreviations

BDL: bile duct ligation
BSA: bovine serum albumin
CCl_4_: carbon tetrachloride
CM: conditioned media
ECM: extracellular matrix
ELISA: enzyme linked immunosorbent assay
HSCs: hepatic stellate cells
LAP: latency-associated protein
LAP β1: TGF-β1 LAP
LAP-DP: LAP degradation product
LOQ: lowest limit of quantification
NASH: non-alcoholic steatohepatitis
PLK: plasma kallikrein
PLN: plasmin
RT-PCR: reverse transcription-polymerase chain reaction
rhLAP β1: recombinant human LAP β1
TGF-β1: transforming growth factor-β1
αSMA: α-smooth muscle actin
