# Supplementary figures and images for: L59 TGF-β LAP degradation products serve as a promising blood biomarker for liver fibrogenesis in mice
Source: Fibrogenesis Tissue Repair. 2015 Sep 15;8:17. doi: 10.1186/s13069-015-0034-9 (PMC4570586; doi:10.1186/s13069-015-0034-9)

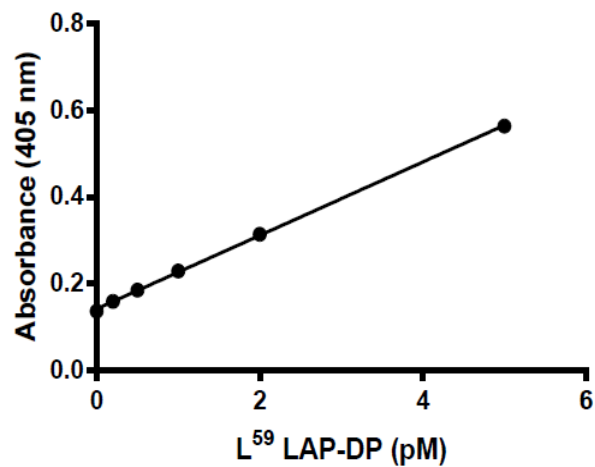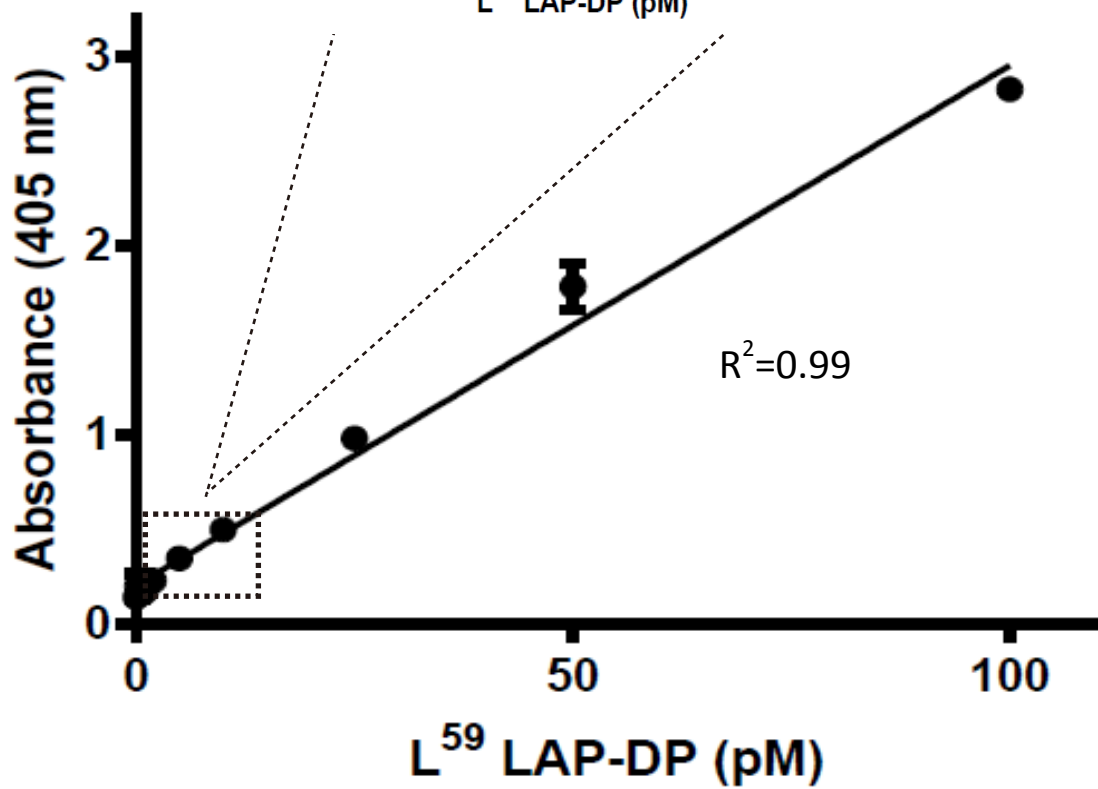

Supplement: Additional file 1: Figure S1. — The standard curve for the L59 LAP-DP ELISA. The absorbance linearly increased up to about 100 pM. The lower limit of quantitation is approximately 2 pM. [file 13069_2015_34_MOESM1_ESM.pdf]

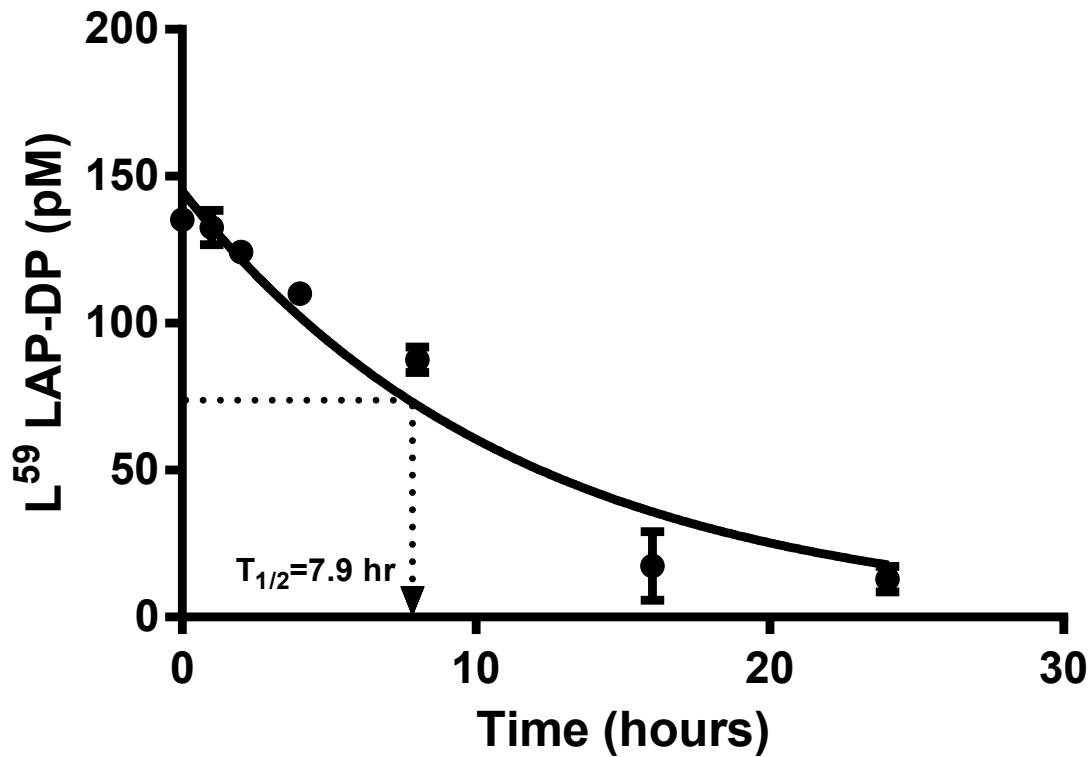

Supplement: Additional file 2: Figure S2. — The half-life of L59 LAP-DPs in mouse plasma. Exogenously generated L59 LAP-DPs were added to mouse plasma and incubated at 37 °C for 0, 1, 2, 4, 8, 16, and 24 h. The remaining levels were measured and plotted against incubation time, and the half-life time was determined. [file 13069_2015_34_MOESM2_ESM.pdf]
